# Observational, Multicenter, Retrospective, Study on the Usage Patterns of the Fixed Dose Combination of Glimepiride, Metformin, and Voglibose in Type 2 Diabetes Management

**DOI:** 10.7759/cureus.52064

**Published:** 2024-01-10

**Authors:** Paramesh Shamanna, Pankaj Kumar Jha, Altaf Makwana, Heta Shukla, Chintan Bavishi

**Affiliations:** 1 Department of Diabetes and Endocrinology, Bangalore Diabetes Centre, Bangalore, IND; 2 Department of Medical Services, Torrent Pharmaceuticals Ltd., Ahmedabad, IND

**Keywords:** usage pattern, voglibose, metformin, glimepiride, fixed dose combination, observational study

## Abstract

Objective

This study aimed to evaluate and analyze the characteristics of Indian patients with type 2 diabetes mellitus (T2DM) in relation to the usage patterns of a fixed-dose combination (FDC) of glimepiride, metformin, and voglibose.

Methods

This retrospective, observational, multicentric analysis was conducted from March 2021 to September 2022. It involved adult patients (aged ≥18 years) with T2DM from 424 sites including a combination of hospitals and privately owned clinics across India to ensure comprehensive representation of the patient population The study included patients who had been treated with FDC of glimepiride, metformin, and voglibose of varying strengths for T2DM management. Data were collected through a pre-designed electronic form, which captured demographic details, medical history, T2DM history, and drug usage patterns from medical records. The collected data were then analyzed using descriptive statistical methods.

Results

This analysis encompassed a final cohort of 8,587 patients out of which 5,840 were males with a mean age of 54.91 years and a BMI of 28.41 kg/m^2^. Newly diagnosed T2DM cases were 35.23%, 54.79% had a family history, and 61.21% had risk factors such as smoking, sedentary lifestyle, and others. Dyslipidemia (13.94%) and neuropathy (14.48%) were common comorbidities. The most prescribed FDC was 1 mg glimepiride, 500 mg metformin, 0.2 mg voglibose (40.14%), the most preferred dosing frequency was once daily (52.92%) and the most common duration of treatment was one to three months (48.78%).

Conclusion

In routine Indian clinical practice, the triple drug FDC of 1 mg glimepiride, 500 mg metformin, and 0.2 mg voglibose, taken once daily for one to three months, was the most common treatment for both newly diagnosed and long-standing diabetes patients.

## Introduction

Diabetes mellitus, particularly type 2 (T2DM), stands as a major health concern globally, affecting approximately 537 million people, with a significant number in low and middle-income countries [1]. In India, the situation is particularly alarming, as indicated by the Indian Council of Medical Research-India Diabetes-17 (ICMR-INDIAB-17) study, which reports 101 million individuals with diabetes and 136 million with prediabetes [2]. The critical goal of diabetes management, as emphasized by pivotal studies, such as the Diabetes Control and Complications Trial (DCCT) and the United Kingdom Prospective Diabetes Study (UKPDS), is to maintain near-normal blood glucose levels to reduce the risk of chronic complications [3,4].

The management of T2DM often requires a multidrug approach due to its complex pathophysiology. However, the individual administration of multiple drugs can lead to poor adherence and inadequate glycemic control [5]. Fixed dose combinations (FDCs) of antidiabetic medications have emerged as a solution, enhancing adherence and simplifying treatment [6,7]. Metformin, a biguanide, is recommended as the first-line pharmacotherapy in T2DM, combined with lifestyle changes. When monotherapy is insufficient, the treatment escalates to dual or triple drug therapies based on Hb1Ac levels [8]. Despite the known effectiveness of the triple drug combination of glimepiride, metformin, and voglibose in controlling glucose levels, there is a notable lack of data regarding the usage patterns of different strengths of this FDC in India's routine clinical practice.

This study addresses this gap by investigating the prescribing patterns of the FDC of glimepiride, metformin, and voglibose among healthcare professionals in India. This study aimed to shed light on the real-world application of this FDC in T2DM management, thereby enriching the understanding of diabetes care in the Indian context.

The abstract of this article has been accepted and will be presented as a meeting abstract at the International Diabetes Federation (IDF) Virtual Congress on December 4-7, 2023.

## Materials and methods

Study design

This retrospective, multicenter, cross-sectional observational analysis was conducted across India from March 2021 to September 2022. The choice of retrospective design was based on its ability to provide a real-world and cost-effective method to evaluate the usage patterns of FDC of glimepiride, metformin, and voglibose in T2DM management. Adhering to ethical standards in line with the Declaration of Helsinki, the International Conference on Harmonization-Good Clinical Practices (ICH-GCP), and relevant non-interventional study laws, this research upheld high ethical integrity. The study's protocol and Data Collection Form (DCF) received approval on March 31, 2021, from the Institutional Ethics Committee of Sangini Hospital, Ahmedabad. Data collection occurred at a single time point, involving no intervention.

Study objectives and inclusion and exclusion criteria

The primary objective of the study was to understand the usage pattern of FDC of glimepiride, metformin, and voglibose in T2DM patients and the secondary objective was to determine demographic distribution, patient characteristics, and prevalence of common comorbid conditions. The eligibility criteria for the patients are mentioned in Table 1.

**Table 1:** Eligibility criteria of study population.

Criteria		Rationale
Inclusion criteria	Age ≥18 years	To ensure that participants included in the study were of adult age
	Patients with T2DM	To guarantee that the individuals included in the study align with the targeted population as outlined in the study's objectives
	Treated with FDC of glimepiride, metformin, and voglibose	To verify that the administered treatment in the study is consistent with the treatment protocol defined for the research
Exclusion criteria	Incomplete data	To ensure the collection of high-quality data in order to fulfill the study objectives
	Investigator considered patient not suitable	Provides flexibility for the investigator to exclude the patients that may compromise the objectives of study or ethical considerations

Data collection

The study sourced data from 424 different sites including a combination of hospitals and privately owned clinics across various regions in India, ensuring a comprehensive and accurate representation of the patient population. To facilitate data collection, electronic data collection forms (eDCFs) were distributed to the participating sites by the study sponsor. Clinicians at these sites were responsible for completing these forms, relying on the medical records of their patients for accurate information. As a cross-sectional study, there was a potential for misclassification attributable to recall bias. Nevertheless, the data were filled by clinicians and cross-verified with patients' medical records to minimize the likelihood of recall bias.

The eDCFs were meticulously designed to gather specific details. To minimize errors, most sections of the forms were structured to allow clinicians to simply mark checkboxes. The information collected encompassed a wide range of data, including demographic details, patient medical history, diabetic complications, comorbidities, and specifics about the fixed-dose combination (FDC) medication, such as dose, dosing frequency, and duration of treatment.

To ensure the integrity and quality of the data collected, several checks were implemented. These included logic consistency checks and verification for extreme or missing values. Only data that passed these rigorous quality standards and checks were considered valid for inclusion in the analysis, ensuring the reliability of the study's findings.

The study commenced with an initial patient pool of 10,824 individuals. The data of 2,237 patients were excluded due to non-availability of complete data. Hence, the study encompassed a final cohort of 8,587 individuals who provided complete data for analysis.

Statistical analysis

The final analysis of this study incorporated the anonymized data from 8,587 enrolled patients. In cases where data for a specific variable was missing, those patients were excluded only from the analysis of that particular variable. This approach ensured that the integrity and comprehensiveness of the dataset were maintained for the majority of the analyses. Microsoft Excel 2016 was used as the tool for conducting basic statistical analyses as intricate statistical methods were not needed for this study. Descriptive statistics, including measures such as mean, percentage, and frequency were computed. The dataset analysis specifically relied on these statistical summaries. By using these methods, the study was able to effectively quantify and summarize the key characteristics and trends within the large dataset.

## Results

Primary outcome measures

Usage Pattern of FDC

**Figure 1:**
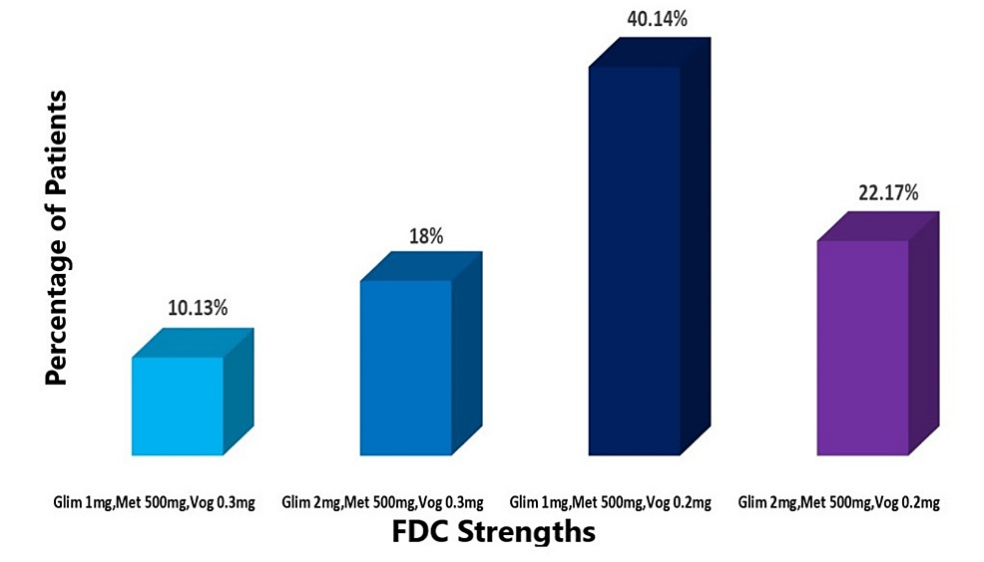
Usage of the various strengths of glimepiride, metformin, and voglibose combinations. Glim: glimepiride; Met: metformin; Vog: voglibose

FDC strength: The most commonly prescribed FDC for treating type 2 diabetes mellitus was glimepiride 1 mg, metformin 500 mg, and voglibose 0.2 mg. This specific strength of FDC was administered to 3,447 patients, accounting for 40.14% of the total cohort. Among the other prescribed FDC strengths glimepiride 2 mg, metformin 500 mg, and voglibose 0.2 mg were given to 1,897 patients (22.17%), glimepiride 2 mg, metformin 500 mg, and voglibose 0.3 mg to 1,544 patients (18.04%), and glimepiride 1 mg, metformin 500 mg, and voglibose 0.3 mg to 867 patients (10.13%). The clear breakdown of the prescription patterns of various FDC strengths in the management of T2DM in the study population is visually represented in Figure 1.

Dosing Frequency

**Figure 2:**
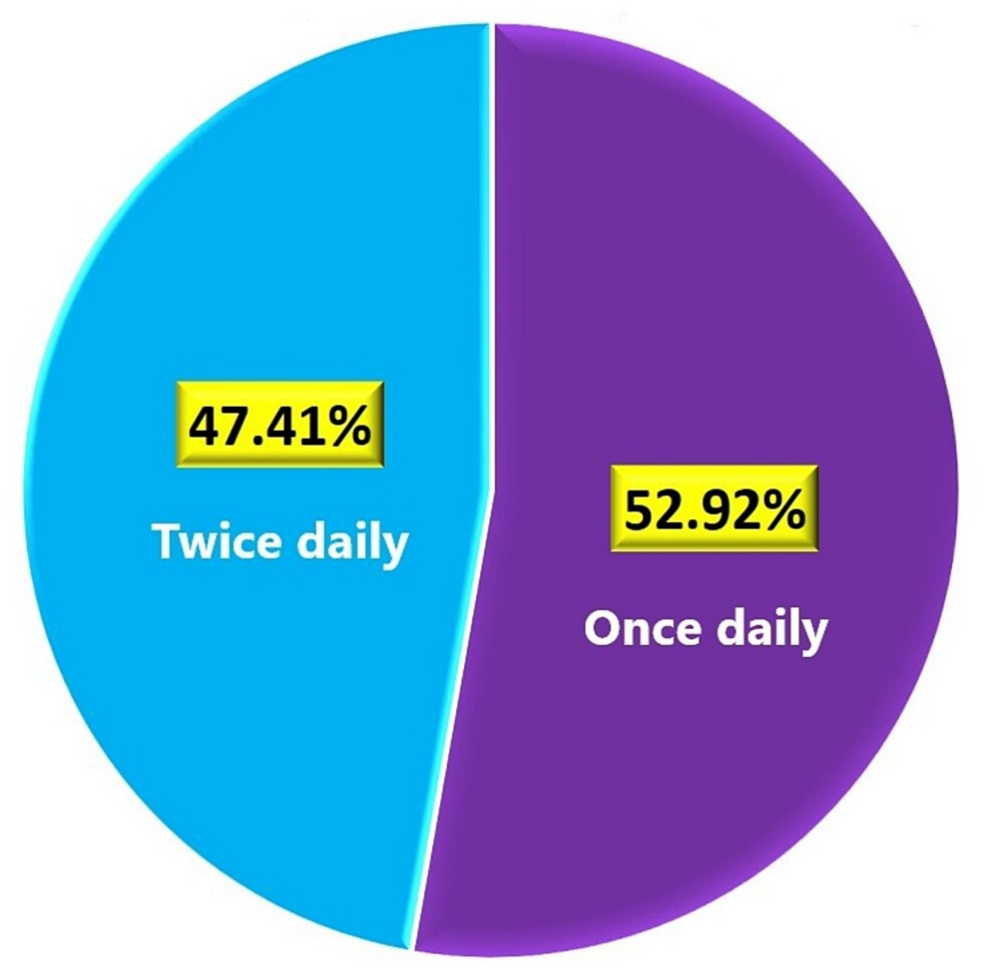
Dose frequency of FDC of glimepiride, metformin, and voglibose. FDC: fixed-dose combination

Among all the patients prescribed with any strength of FDCs, 4,529 (52.92%) had once-daily dosing, while 4,058 (47.41%) had twice-daily dosing as illustrated in Figure 2.

Treatment Duration

**Figure 3:**
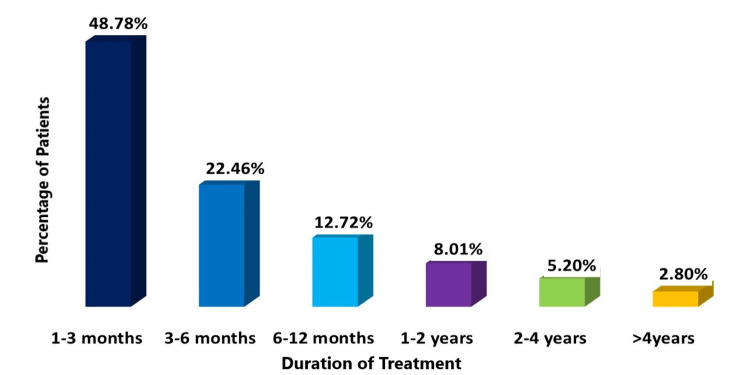
Treatment duration of FDC of glimepiride, metformin, and voglibose. FDC: fixed-dose combination

The most common treatment duration for patients prescribed with fixed-dose combinations (FDCs) was one to three months encompassing 4,189 patients or 48.78%. Notably, other durations included three to six months (1,929 patients or 22.46%), six to 12 months (1,093 patients or 12.72%), one to two years (688 patients or 8.01%), two to four years (447 patients or 5.2%), and >4 years (241 patients or 2.8%) (Figure 3).

Secondary outcome measures

Demographic Distribution

A higher percentage of male patients with T2DM was observed, comprising 68%, while females accounted for 32%. The average age of the patient cohort was 54.91 years, and the mean body mass index (BMI) for the study population was 28.41 kg/m^2^ (Table 2).

**Table 2:** Demographic distribution of the study population. BMI: body mass index

Population characteristics	
Age (years, mean)	54.91
Male, n (%)	5,840 (68%)
Female n (%)	2,747 (32%)
BMI (kg/m^2^, mean)	28.41

Patient Characteristics

More than half (4,706 or 54.79%) of the patients had a family history of diabetes. A substantial portion (3,026 or 35.23%) were newly diagnosed diabetes cases, while others had diabetes history of one to three years (2,768 or 32.22%), three to six years (1,812 or 21.09%), and >6 years (981 or 11.44%). The sedentary lifestyle was reported by 3,189 (37.13%) patients as most common risk factor followed by smoking in 2,022 (23.54%), alcohol use in 1,547 (18.01%), and obesity in 750 (8.73%) cases. The most common diabetic complications were neuropathy and nephropathy, affecting 1,244 (14.48%) and 767 (8.93%) persons, respectively (Table 3).

**Table 3:** Risk factors and complications associated with T2DM. T2DM: type 2 diabetes mellitus

Patient characteristics	Particulars	N	%
Family history of diabetes	Yes	4,706	54.79%
	No	3,881	45.21%
Duration of diabetes (years)	Newly diagnosed	3,026	35.23%
	1-3	2,768	32.22%
	3-6	1,812	21.09%
	>6	981	11.44%
Identified risk factor	Sedentary lifestyle	3,189	37.13%
	Smoking	2,022	23.54%
	Alcohol use	1,547	18.01%
	Obesity	750	8.73%
Common diabetic complication(s)	Neuropathy	1,244	14.48%
	Nephropathy	767	8.93%

Common Comorbid Conditions

The most prevalent comorbidity among patients with T2DM was dyslipidemia, reported in 1,197 or 13.94% of patients. Additionally, kidney disease was observed in 605 or 7.04% of patients, while hypertension was identified as a comorbidity in 1.03% of T2DM cases (Table 4).

**Table 4:** Prevalence of common comorbid conditions of T2DM. T2DM: type 2 diabetes mellitus

Comorbid conditions	N	%
Dyslipidemia	1,197	13.94%
Kidney disease	605	7.04%
Hypertension	89	1.03%

## Discussion

We analyzed the usage pattern of various strengths of fixed-dose combinations (FDCs) for T2DM management. The most commonly prescribed FDC was glimepiride 1 mg, metformin 500 mg, and voglibose 0.2 mg, administered to 3,447 patients (40.14% of the total cohort). Other prescribed combinations included metformin 500 mg with higher doses of glimepiride and voglibose, catering to a significant portion of the study population. In terms of dosing frequency, over half of the patients (52.92%) were on once-daily dosing, while the remainder (47.41%) followed a twice-daily regimen. The majority of patients (48.78%) underwent treatment with FDC for one to three months, with varied durations observed in the remaining participants.

The use of a triple-drug combination comprising glimepiride, metformin, and voglibose has demonstrated its effectiveness in controlling both fasting and postprandial blood glucose levels in patients with T2DM [9]. This FDC has demonstrated several favorable outcomes, such as adequate glycemic control, weight reduction, and no tolerability concerns in Indian patients with T2DM [10].

In this retrospective observational study, we explored the extensive prescription of various strengths of the FDC of glimepiride, metformin, and voglibose across India. This widespread use highlights the perceived efficacy and cost-effectiveness of this combination in managing T2DM. Our analysis provides valuable insights into the usage patterns of these FDC strengths in T2DM patients.

The study's findings, particularly the predominant preference for glimepiride 1 mg, metformin 500 mg, and voglibose 0.2 mg FDC, underscore its effectiveness in glycemic control. This preference suggests that a balanced combination of lower medication doses can achieve therapeutic goals while minimizing side effects. The observed preference for once-daily dosing, noted in our study, underscores the importance of patient convenience and treatment adherence. Simplified regimens, by reducing medication complexity, may enhance adherence and, consequently, treatment efficacy.

However, it is crucial to recognize the individual variability in treatment response among patients. Healthcare providers must consider patient-specific factors, glycemic control goals, previous medication history, and side effects profile when determining the most appropriate FDC strength and treatment duration [9]. The duration of treatment in clinical practice involves a delicate balance between optimizing therapeutic benefits, managing side effects, and the unique characteristics of the patients.

The substantial sample size of 8,587 patients in our study provides a robust dataset, enhancing the reliability and representativeness of our findings. The global prevalence of diabetes is higher in males. Our study also sheds light on the demographic distribution of diabetes, confirming a higher prevalence among Indian males, and aligning with broader epidemiological trends [11]. Most people with diabetes are diagnosed at ages 45-64 years. The mean age of the patients in this study was 54.91 years, fitting within the typical onset age range for diabetes (45-64 years) [12]. Elevated BMI is consistently associated with an increased risk of T2DM. In our study, the mean BMI of 28.41 kg/m^2^ was higher than average, emphasizing the well-known link between increased BMI and diabetes risk [13].

The study also highlighted that 35.23% of the participants were newly diagnosed patients. This finding emphasizes the importance of early diagnosis and intervention for optimal glycemic control and long-term outcomes. The significant proportion of patients with a positive family history of diabetes (54.79%) reinforces the genetic predisposition to T2DM [14]. This necessitates targeted screening and preventive measures for individuals with a family history of diabetes.

Moreover, the presence of other risk factors like smoking and sedentary lifestyles underlines the multifactorial nature of T2DM. The history of smoking among diabetic patients in our study and the association with increased diabetes risk align with established research on the impact of smoking on insulin resistance and T2DM development [15]. Similarly, our findings regarding physical activity align with numerous studies demonstrating its inverse relationship with T2DM risk [16]. Modifiable risk factors like smoking and sedentary lifestyle significantly influence T2DM development. Lifestyle interventions and regular physical activity play key roles in the management of T2DM. Furthermore, the identification of dyslipidemia as the most common comorbidity in our cohort corroborates the well-established connection between diabetes and dyslipidemia. With the increasing duration of T2DM, insulin resistance increases which consequently influences carbohydrate and lipid metabolism [17]. The prevalence of microvascular complications, particularly neuropathy, reflects the well-documented association between diabetes and these complications [18].

Our study emphasizes the tailored prescription of FDC strengths to manage T2DM effectively, highlighting the importance of dose titration for optimal glucose control. The large sample size of this study strengthens the reliability of the data, making it generalizable to the broader population. Nevertheless, the retrospective nature of our study introduces limitations such as potential recall bias, incomplete records, and variations in data collection practices over time. Lack of randomization and control may introduce confounding variables, challenging the establishment of causal relationships.

While retrospective studies offer valuable insights, prospective studies provide a more controlled framework for the generation of high-quality data. Combining findings from both study types contributes to a more comprehensive understanding of the scientific question under investigation. This limitation highlights the need for further research, including prospective studies or randomized clinical trials, to evaluate the findings and explore causal relationships.

## Conclusions

In conclusion, this study provides critical insights into the prescribing patterns of fixed-dose combinations (FDCs) of glimepiride, metformin, and voglibose for T2DM management in India. The widespread preference for the FDC containing glimepiride 1 mg, metformin 500 mg, and voglibose 0.2 mg, as evident in our large sample size of 8,587 patients, highlights its effectiveness in glycemic control. This finding indicates the efficacy of using lower medication doses for managing T2DM with minimal side effects. These insights may have the potential to significantly impact clinical practice, promoting a more personalized and adaptable approach to diabetes management in India. While the retrospective nature of the study limits the ability to establish causal relationships, the insights gained are invaluable for guiding future research and clinical practice. Future research on causal relationships, treatment outcomes, and comparative effectiveness of FDC strengths will refine understanding and contribute to evidence-based guidelines for clinicians. This study contributes significantly to the understanding of diabetes management in India and provides a foundation for optimizing treatment strategies for T2DM patients.
